# Anguimorpha as a model group for studying the comparative heart morphology among Lepidosauria: Evolutionary window on the ventricular septation

**DOI:** 10.1002/ece3.9476

**Published:** 2022-11-08

**Authors:** Martina Gregorovicova, Martin Bartos, Bjarke Jensen, Jiri Janacek, Bryan Minne, Jiri Moravec, David Sedmera

**Affiliations:** ^1^ First Faculty of Medicine, Institute of Anatomy Charles University Prague Czech Republic; ^2^ First Faculty of Medicine Institute of Dental Medicine, Charles University Prague Czech Republic; ^3^ Department of Medical Biology, Amsterdam Cardiovascular Sciences University of Amsterdam Amsterdam The Netherlands; ^4^ Laboratory of Biomathematics, Institute of Physiology Czech Academy of Sciences Prague Czech Republic; ^5^ Amphibian Evolution Lab Free University of Brussels Brussels Belgium; ^6^ National Museum Prague Czech Republic; ^7^ Laboratory of Developmental Cardiology, Institute of Physiology Czech Academy of Sciences Prague Czech Republic

**Keywords:** evolutionary traits, *Lanthanotus*, *Salvator*, septation, *Sphenodon*, *Varanus*, ventricle

## Abstract

The group Anguimorpha represents one of the most unified squamate clades in terms of body plan, ecomorphology, ecophysiology and evolution. On the other hand, the anguimorphs vary between different habitats and ecological niches. Therefore, we focused on the group Anguimorpha to test a possible correlation between heart morphology and ecological niche with respect to phylogenetic position in Squamata with *Sphenodon*, *Salvator*, and *Pogona* as the outgroups. The chosen lepidosaurian species were investigated by microCT. Generally, all lepidosaurs had two well‐developed atria with complete interatrial septum and one ventricle divided by ventricular septa to three different areas. The ventricles of all lepidosaurians had a compact layer and abundant trabeculae. The compact layer and trabeculae were developed in accordance with particular ecological niche of the species, the trabeculae in nocturnal animals with low metabolism, such as *Sphenodon*, *Heloderma* or *Lanthanotus* were more massive. On the other hand athletic animals, such as varanids or *Salvator*, had ventricle compartmentalization divided by three incomplete septa. A difference between varanids and *Salvator* was found in compact layer thickness: thicker in monitor lizards and possibly linked to their mammalian‐like high blood pressure, and the level of ventricular septation. In summary: heart morphology varied among clades in connection with the ecological niche of particular species and it reflects the phylogenetic position in model clade Anguimorpha. In the absence of fossil evidence, this is the closest approach how to understand heart evolution and septation in clade with different cardiac compartmentalization levels.

## Graphical abstract

All lepidosaurians have ventricles composed of the compact layer as well as the trabeculae. Proportion between compact wall and trabeculae differs among squamates in correlation with their physiological needs and ecological niche. Phylogeny and ecological niche are reflected on heart morphology in the group Anguimorpha.

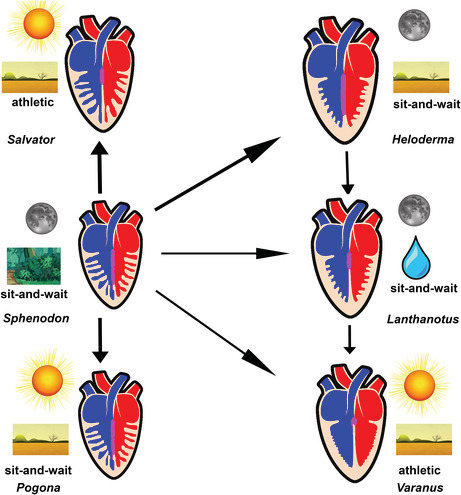

## 1 INTRODUCTION

After more than one century of studying the cardiovascular system in tetrapods, the sauropsid hearts still fascinate the scientists because of the cardiac chambers variable arrangement. Great variation in the heart development and morphology in sauropsids are well described (Hanemaaijer et al., 2019; Jensen et al., 2014; Kvasilova et al., 2018). However, the reasons underlying these variations are poorly understood. In extant sauropsids, the following major lineages are described (Tzika et al., 2011): crocodylians together with birds—archosaurs—crown group (Brusatte et al., 2010), turtles, crocodylians, and birds—archelosaurs—recently proposed and formalized clade (Chiari et al., 2012; Crawford et al., 2015; Joyce et al., 2021; Simões et al., 2022). The last lineage is lepidosaurs, which consist of two clades—squamates and tuataras (Hedges & Poling, 1999). From cardiovascular point of view, the level of the heart septation ranges among these lineages from single undivided ventricle in turtles and the most of the lepidosaurians to fully‐septated ventricle in crocodylians and birds (Jensen & Christoffels, 2020; Koshiba‐Takeuchi et al., 2009). A complete septation, resulting in four chambers, is found only in crocodylians, birds, and mammals (Poelmann et al., 2014; Poelmann & Gittenberger‐de Groot, 2019). Thus, lepidosaurian hearts are less studied than the hearts of archosaurs and mammals (Holmes, 1975; Summers, 2005) because of mostly undivided ventricle. Hence, lepidosaurian cardiovascular system and its evolution are still unresolved.

Typically, lepidosaurian heart consists of two well‐developed atria with a complete septum, and a single ventricle. Three incomplete septa, muscular ridge, bulbuslamelle and vertical septum divide the ventricle into cavum venosum, cavum pulmonale, and cavum arteriosum (Jensen et al., 2014) for a better blood separation into two circuits—pulmonary and systemic one (Hicks, 2002). In addition, in lepidosaurians three great vessels arise from the ventricle—pulmonary artery, and the left and right aortic arches (Jensen, Nyengaard, et al., 2010). Moreover, the pulmonary development and morphology (Perry, 1998), oxygen consumption (Crossley & Burggren, 2009), and therefore the aerobic metabolism (Hillenius & Ruben, 2004) are connected not only to body size and body temperature but also behavior, ecology, and life histories play a role. From physiological point of view, the maximum oxygen consumption in squamates is linked to active foraging and to carnivorous lineages such as Varanidae and Helodermatidae (Albuquerque & Garland Jr, 2020). All things considered, all these significant features are reflected on cardiovascular system and they have a great impact on the lepidosaurian heart anatomy and physiology.

However, little is known about the correlation of the heart morphology, ecological niche and phylogenetic position among lepidosaurs (Harmon et al., 2005; Vitt & Pianka, 2005). Convergence of a particular ecological niche as it is observed e.g. between genus *Salvator* (Teiidae) and *Varanus* (Varanidae; Cechin et al., 2011; Pianka & King, 2004) could be reflected on functional heart morphology among squamates as well. Therefore, lepidosaurs are good models for testing evolutionary hypotheses with focusing on adaptive ecomorphology and ecophysiology (Camargo et al., 2010) also from the cardiac point of view (Jensen & Christoffels, 2020; Moorman & Christoffels, 2003).

**FIGURE 1:**
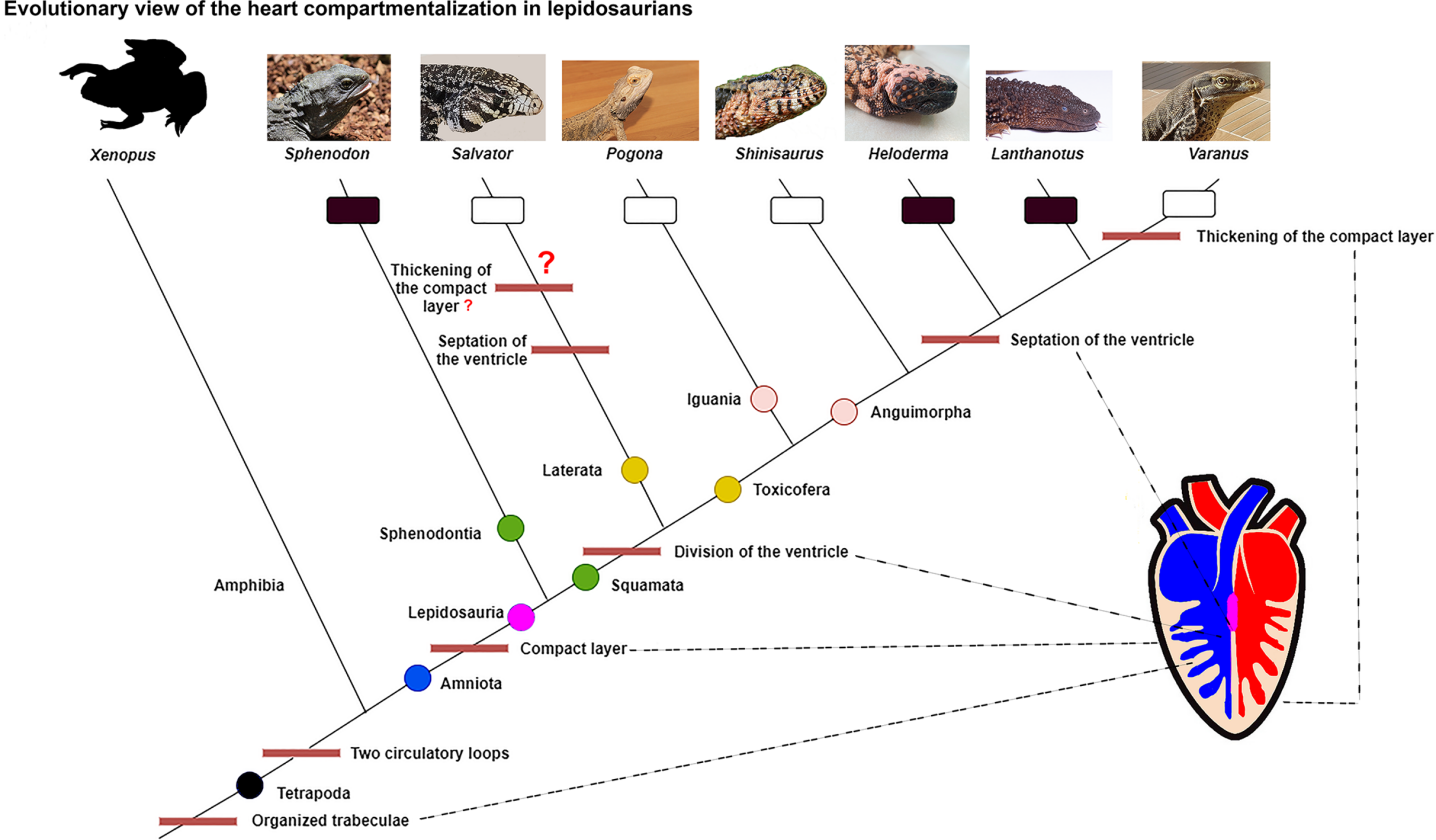
Evolutionary view of the heart compartmentalization in lepidosaurs with focusing on key heart morphological features (red lines), how they appear across the phylogenetic tree. The question mark indicates possible heart features appearance of convergence between genus *Salvator* and *Varanus*. Black box—nocturnal species; white box—diurnal species; black circle—Tetrapoda; blue circle—Amniota; magenta circle—Lepidosauria; green circles—branching lepidosaurs to Sphenodontia and Squamata; yellow circles—inner simplified branching squamates to Laterata and Toxicofera; red circles—inner simplified branching of Toxicofera to Iguania and Anguimorpha, according to Pyron et al. (2013), Wiens et al. (2012). Chosen animal photographs were provided by Martina Gregorovicova (*Sphenodon, Salvator, Shinisaurus, Heloderma, Pogona*, and *Varanus*), and Laura Ruysseveldt (*Lanthanotus*).

The following important features that impacted the heart evolution across the phylogenetic tree (Figure 1) are: ventricle division to specific cava by trabeculae, ventricular septation and compact layer thickening.

The compact layer presence and thickening are particularly important characteristics in terms of efficient blood circulation through the animal body (Farrell et al., 1998; Sedmera et al., 2000). Moreover, the compact layer development goes hand in hand with the occurrence of coronary arteries, which supply the myocardium with blood (Oštádal, 1999). Aerobic activity and also ecological niche are important drivers of such compact myocardial development and thickening during cardiac phylogenesis in vertebrates (Genge et al., 2012). Therefore there is no surprise that the compact layer as well as the coronary arteries are common features also in reptiles (Hagensen et al., 2008; MacKinnon & Heatwole, 1981; Simons, 1965).

Chosen lepidosaurian species were investigated by microCT across the phylogenetic tree. Firstly, *Sphenodon punctatus* (Sphenodontia) was chosen because it is the closest relative of squamate reptiles (Jones et al., 2013) with very slow metabolism and with relation between metabolic rate and e.g. body mass or temperature giving a metabolic scaling exponent of 0.62, while this exponent ranges from 0.51–0.80 (the higher the number the higher oxygen consumption) in lepidosaurs depending on the species (Andrews & Pough, 1985). Metabolic scaling exponent describes how metabolic rate develops with changes in body mass/temperature and *Sphenodon* has similar metabolic demands as most of the squamates (Thompson & Daugherty, 1998). *Sphenodon* is traditionally classified as a nocturnal (Gillingham & Miller, 1991) and a truly cryophilic reptile (Wells et al., 1990), which has an impact on the cardiovascular system. Such cardiovascular system is generally described as primitive with remains of conus arteriosus. This structure is reduced during heart vertebrate phylogenesis in Sauropsida and in Amniota. It generally means that without conus arteriosus the great arteries are connected directly to the ventricle (Farmer, 2011). The other features are low level of septation and almost no compact layer. As a second outgroup to anguimorphs was chosen *Salvator merianae* (Teiidae) because this species occupies a similar ecological niche as varanids (Cechin et al., 2011) and tegus are also known for their seasonal endothermy linked to the reproduction (Tattersall et al., 2016). However, the heart morphology deviates from genus *Varanus* (Hanemaaijer et al., 2019; Jensen et al., 2014) and also the physiology is different from varanids, particularly when comparing blood pressure, which is almost double in favor of Varanidae (12 kPa, mammalian‐like) than of Teiidae (5.66 kPa, typical lizard; Filogonio et al., 2020; Millard & Johansen, 1974; Thompson & Withers, 1997). Moreover, there is no such high oxygen consumption level in Teiidae as in varanids and helodermatids (Albuquerque & Garland Jr, 2020), although the species in the group Teiidae are described mostly as active foragers similarly to varanids. *Pogona vitticeps* (Agamidae) was chosen as the last outgroup, and a member of the crown group Toxicofera with closer phylogenetic relation to Anguimorpha than the group Teiidae (Pyron et al., 2013) but with a different ecological niche (Köhler et al., 2003), especially in comparison to varanids (Pianka & King, 2004) and at the same time being heliothermic animal (Seebacher & Franklin, 2001) in contrast to *Sphenodon*. The group Anguimorpha represents one of the most unified squamate clade (Mesquita et al., 2016; Pianka, 1995; Pianka & King, 2004), which means successful uniform basic body plan, especially in varanids (Ast, 2001; Pianka & King, 2004). The goal of this study was to test the evolutionary hypothesis among the ventricular septation, ecological niche, and phylogenetic position in group Anguimorpha with several outgroup species. The results could help us estimate when and how pressure separation evolved in squamates.

## 2 MATERIALS AND METHODS

Hearts samples were collected from animals freshly dead by senescence from private breeders (*Pogona vitticeps, Shinisaurus crocodilurus*, *Lanthanotus borneensis, Varanus panoptes horni, Varanus acanthurus*) as well as from the specimens held in the herpetological collection of the National Museum, Prague (*Sphenodon punctatus* NMP6V 376514; *Salvator merianae* NMP6V 71376; *Heloderma suspectum* NMP6V 34506; *Varanus griseus* NMP6V 72729/3). For using microCT, all hearts were contrasted in iodine solution from period of 1 week (the smallest samples) to 1 month (the biggest hearts) and in larger specimens additional contrasting through intraventricular injection of the iodine solution was used (Metscher, 2009). The specimens were scanned in plastic tube immersed in 70/96% ethanol according to size and origin of the sample, with the following scanning parameters: 6–16 μm pixel size, camera binning 2 × 2–3 × 3, 0.25 mm, Al filter, frame averaging of 2 and the use of 180° rotation. Scans were acquired using SkyScan 1272 (Bruker, Belgium) and microPET/CT scaner Albira (Bruker, Belgium). Projection images were reconstructed with NRecon (Bruker) with the adequate setting of correction parameters (misalignment, smoothing, ring‐artifact correction and beam hardening). Cross‐sectional images of the scaffolds were provided by DataViewer (Bruker). 3D visualization was created by CT Vox (Bruker), and CTAn (Bruker) was used to perform image processing. Amira Software (Thermo Fisher Scientific) was used for further analysis and calculations. The measurements were obtained from frontal section in the middle part perpendicular to the ventricle. Transversal sections for measuring the volume area between muscular ridge and whole ventricle were obtained by Amira in the upper part of the ventricle (under the atrioventricular canal) and they were measured by using ImageJ software.

## 3 RESULTS

### 3.1 Heart description in selected lepidosaurian species

**FIGURE 2:**
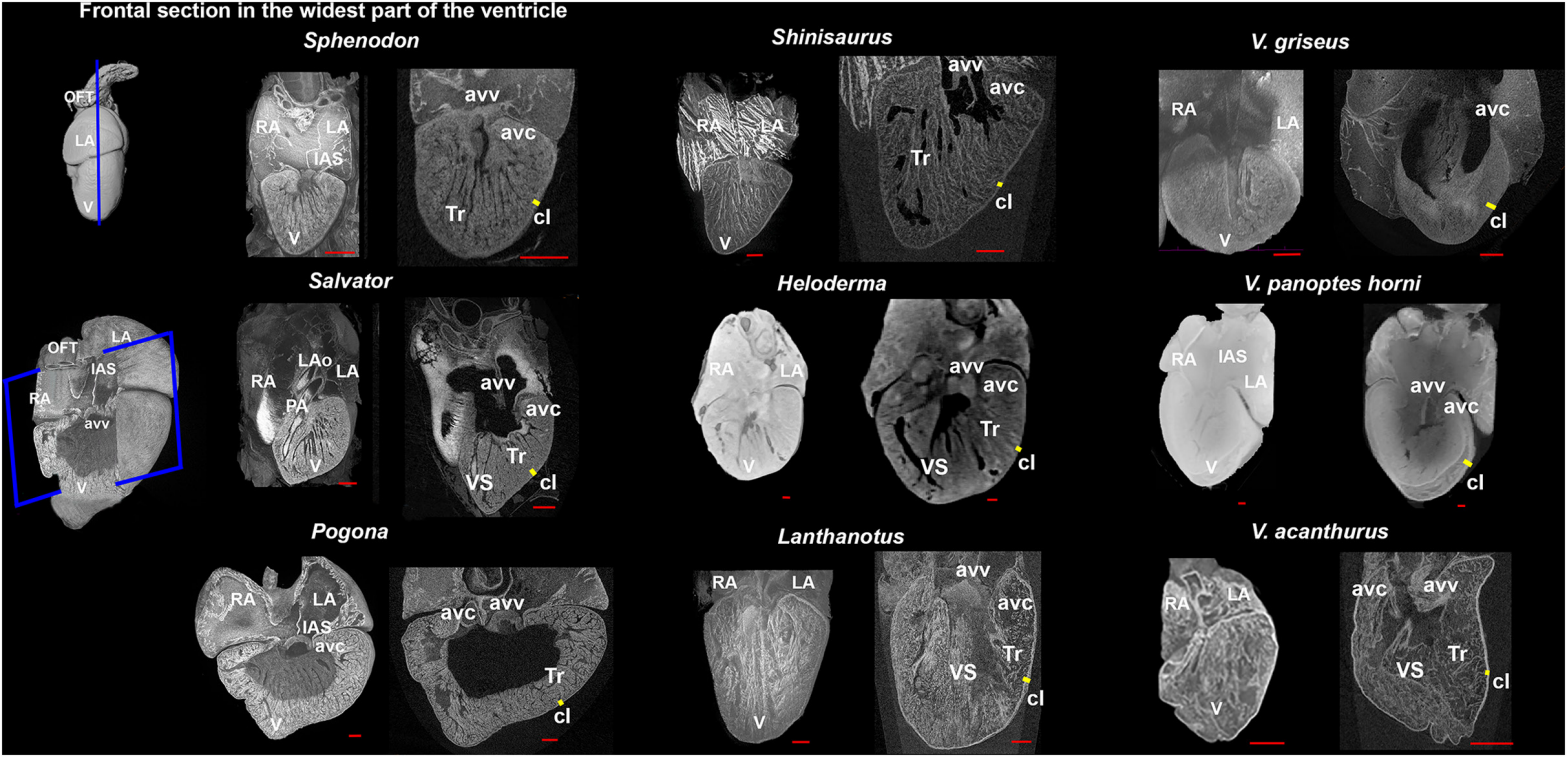
Comparative heart morphology of tested lepidosaurian species with focusing on ventricular septation, trabeculae, and compact layer. The heart frontal sections were performed in the widest part of the ventricle where the septa were fully visible if they occurred. Yellow line—occurrence of the compact layer; red line—scale bar = 1 mm. avc, atrioventricular canal, avv, atrioventricular valve, cl, compact layer, IAS, interatrial septum, LA, left atrium, LAo, left aortic arch, OFT, outflow tract, PA, pulmonary artery, RA, right atrium, Tr, trabeculae, V, ventricle, VS, vertical septum.

**FIGURE 3:**
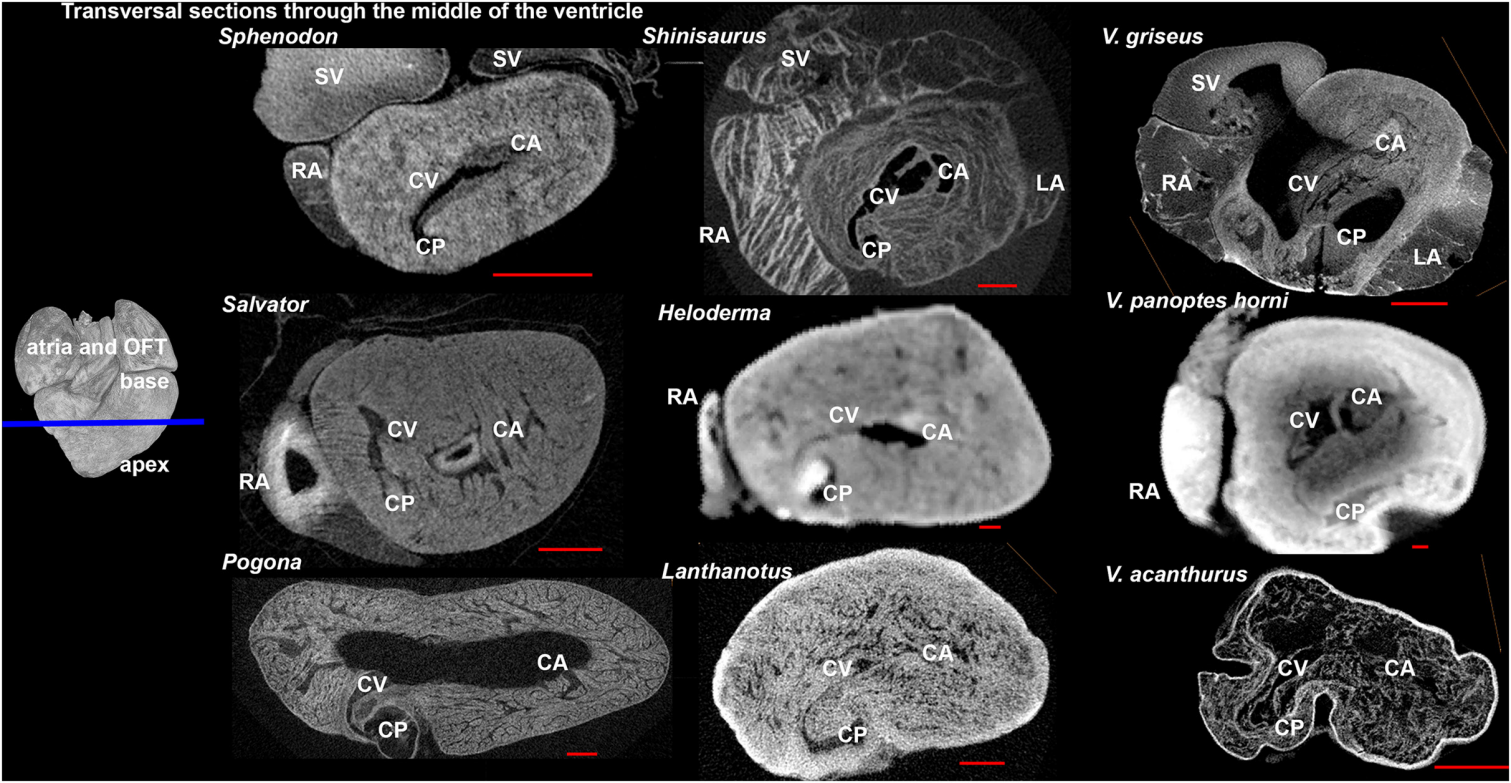
Ventricular cava of chosen lepidosaurian species in transversal sections. The heart sections were performed in the widest part of the ventricle (blue line) where the cava were fully visible. Sections showed position of the ventricular cava. All three cava were presented in all species. Red line—scale bar = 1 mm. CA, cavum arteriosum; CP, cavum pulmonale; CV, cavum venosum; LA, left atrium; RA, right atrium; SV, sinus venosus.

**FIGURE 4:**
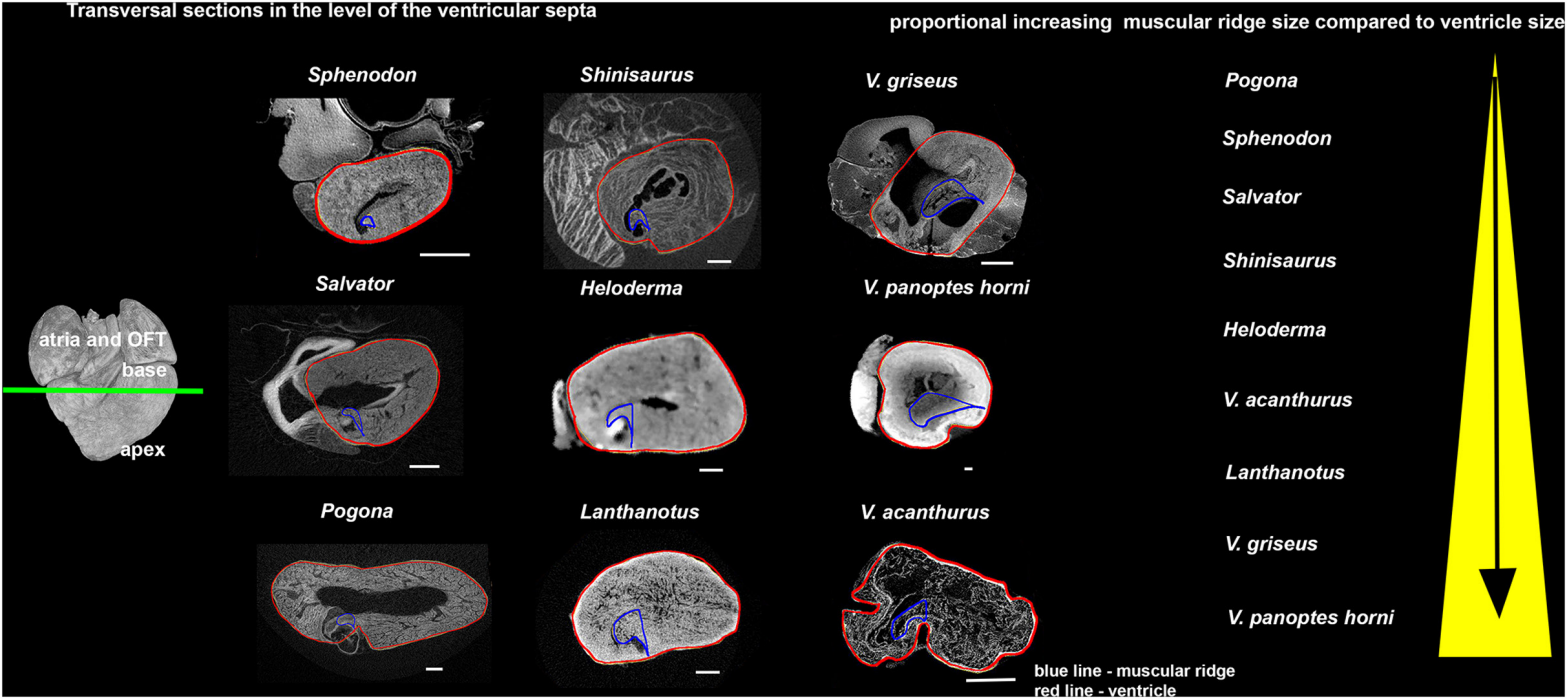
Muscular ridge mass in comparison to ventricular mass in transversal sections close to the heart base (green line) where the muscular ridge was occurred. White line—scale bar = 1 mm; red line—area of the ventricle; blue line—area of the muscular ridge; yellow arrow—increasing mass of the muscular ridge compared with the ventricle. The lowest muscular ridge prominence was found in *Pogona* and *Sphenodon* in comparison to *Lanthanotus* and two large varanids species, *Varanus griseus* and *V. panoptes horni*.

**FIGURE 5:**
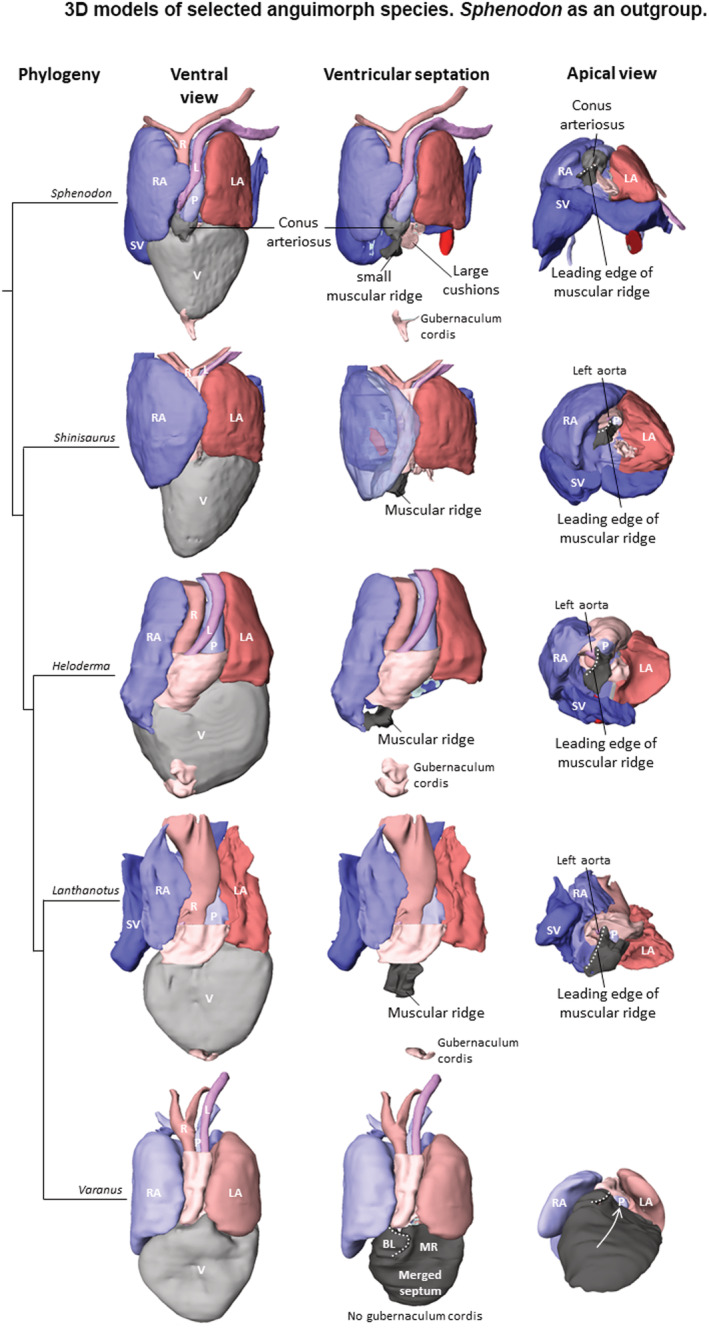
3D models of selected anguimorphs species. *Sphenodon* as an outgroup and deputy species in terms of typical lizard heart morphology in comparison to group Anguimorpha. In *Sphenodon* was observed large conus arteriosus in contrast to muscular ridge, which was very small. *Heloderma* and *Lanthanotus* as the closest monitor lizard relatives had a typical lizard heart morphology and there was observation of the gubernaculum cordis, which helped aligned the heart in the abdominal cavity. In varanids, the ventricular septation was almost completed and the muscular ridge was big in comparison to absence of conus arteriosus. Moreover, there was no occurrence of the gubernaculum cordis in varanids.

**FIGURE 6:**
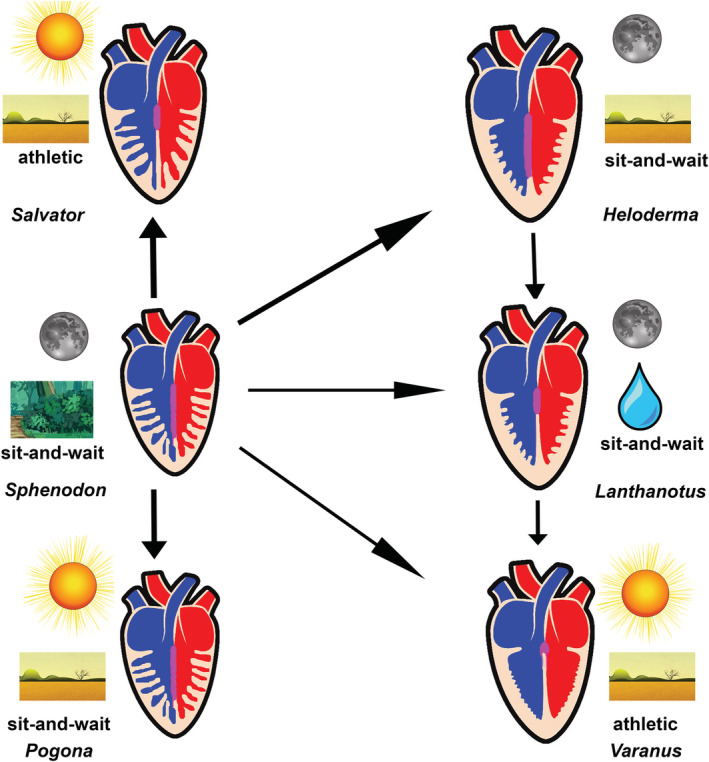
Graphical conclusions of heart compartmentalization among lepidosaurs. Blue—deoxygenated blood; red—oxygenated blood; magenta—mixed blood; sun—diurnal species; moon—nocturnal species; drop—aquatic species; landscape—terrestrial species; forest—forest species; sit‐and‐wait—ambush predator; athletic—active forager.

Generally, all lepidosaurian species had two well‐developed atria with complete interatrial septum, one ventricle divided by three ventricular septa (bulbuslamelle, muscular ridge, and vertical septum) —Figures 2, 3, and by trabeculae to three different cava (cavum venosum, pulmonale, and arteriosum)—Figure 4, a distinct compact layer (Figure 3 and Table 1), and specific area of the muscular ridge in comparison to the ventricular mass—Figure 4 and Table 1. The 3D models morphologically compared the hearts among species in group Anguimorpha with *Sphenodon* as an outgroup (Figure 5). The results revealed that presence of conus arteriosus was the most prominent in *Sphenodon* and it could be counted as a characteristic of primitive heart. The adaptations of selected species, which were reflected on heart morphology, are summarized in Figure 6.

**TABLE 1:** Morphological proportional heart measurements of selected lepidosaurian species.

Species	Ratio between LV/RV compact layer	Ratio septum‐trabeculae/ventricular length	Ratio length/width of ventricle	Ratio muscular ridge/ventricle area
*Sphenodon punctatus*	1.1	0.42	1.04	1.2
*Salvator merianae*	1.19	0.69	1.25	1.5
*Pogona vitticeps*	1.17	NA	0.66	1.1
*Shinisaurus crocodilurus*	1.07	0.4	1.17	1.6
*Heloderma suspectum*	1.57	0.5	0.88	1.9
*Lanthanotus borneensis*	1.57	0.7	1.18	5.6
*Varanus griseus*	1.8	0.8	1.3	8
*Varanus panoptes horni*	1.7	0.6	1.05	11.1
*Varanus acanthurus*	1.5	0.6	1.23	3.5

*Note*: Heart measurements were performed in the widest size of the ventricle. The number between right and left compact layer showed proportional ratio in favor to the left part of the ventricle. The higher number the bigger difference between right and left ventricular part. The biggest difference was observed for varanids where the left part of the ventricle was thickest in tested species. The number between septum/trabeculae and ventricular length showed proportional ratio. The higher number closer to one the better developed level of ventricular septation was observed. The highest septation level was observed in genus *Varanus* where the numbers showed almost complete septation. The high ratio was also observed for *Lanthanotus* and *Salvator*. The length/width ventricular ratio described the heart elongation. The lower number the more round heart. Almost rounded hearts were observed in *Pogona* and *Heloderma*. The ratio between muscular ridge and the ventricular area described the size of the muscular ridge. The higher number the more massive muscular ridge septation. The highest number was gained from genus *Varanus*, especially in V. panoptes horni, and *Lanthanotus*.

Abbreviations: LV, compact layer in left part of the ventricle; NA, data not available due to scanning artifacts; RV, compact layer in right part of the ventricle.

**FIGURE 7:**
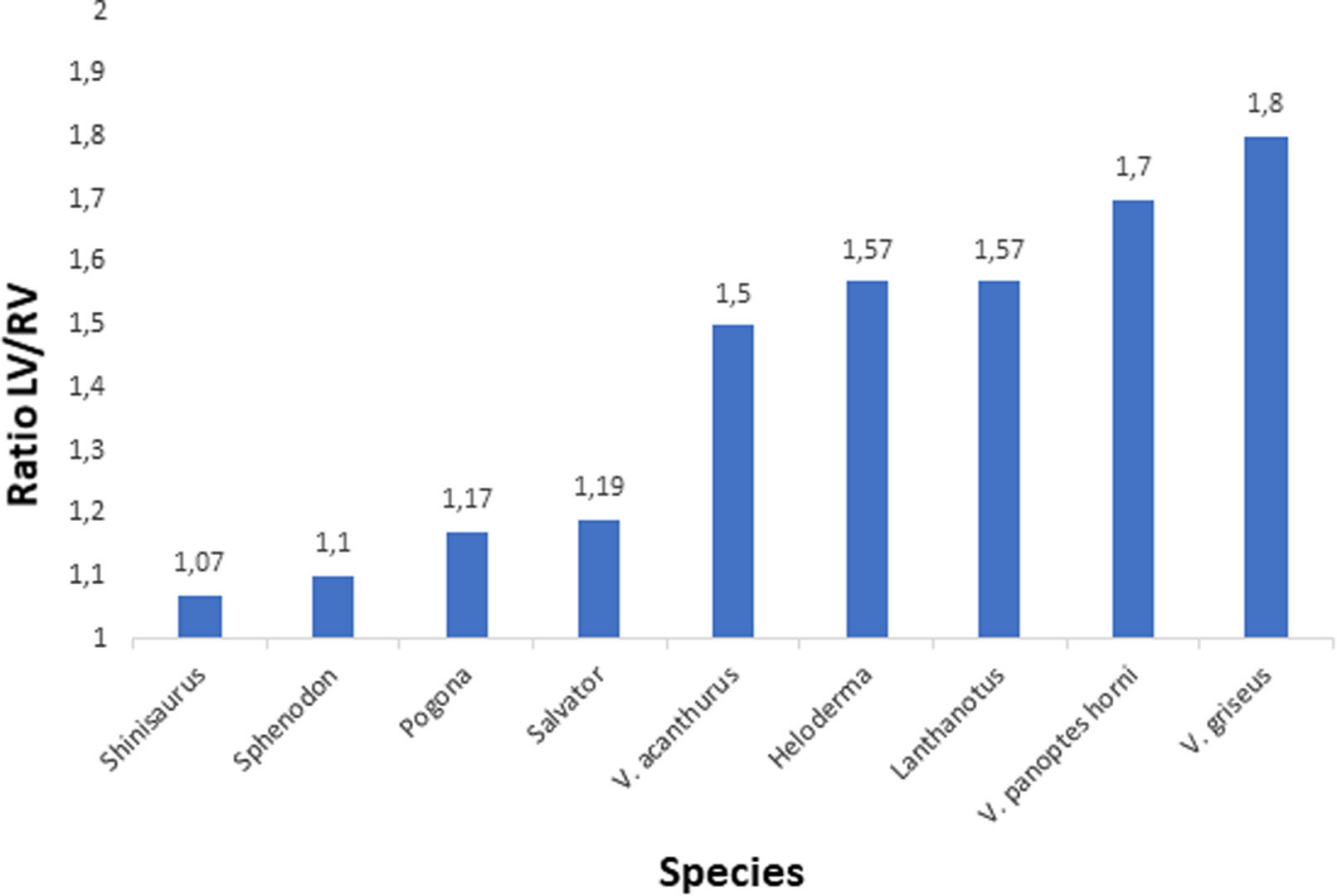
Comparison of the ratio between the left and right ventricular compact layer. Data from Table 1.

**FIGURE 8:**
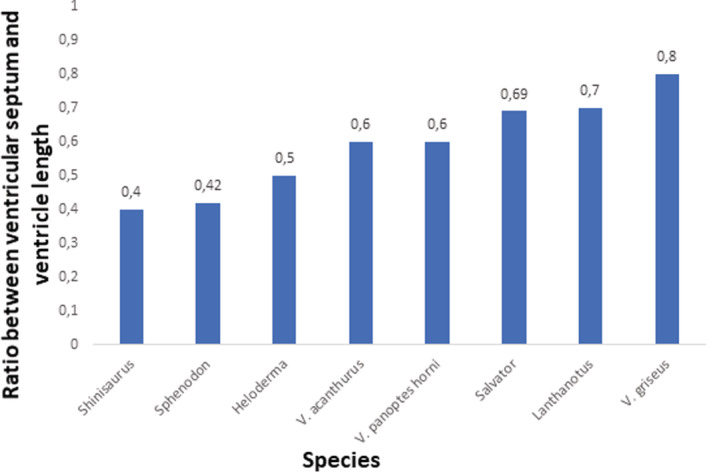
Comparison of the ratio of the ventricular septum/trabeculae length and ventricle length. Data from Table 1. NA, not available.

**FIGURE 9:**
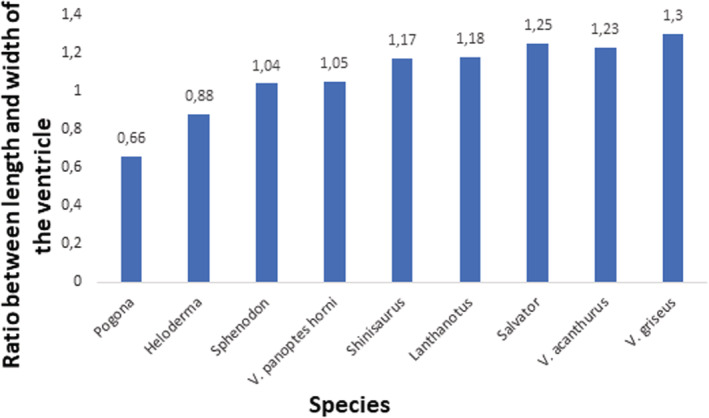
Comparison of the ratio of ventricular length and width. Data from Table 1.

Such adaptations had divergences also among species in the level of the ventricular septation in comparison to the whole ventricular length, or in different proportions between ventricular length and width (Table 1 and Figures 7, 8, 9). More distinct vertical septum was presented in diurnal active foragers, such as in varanids. Another difference was found in the thickness of the compact layer, which was also better developed in favor of mainly diurnal and active species. Specific characteristics found in particular lepidosaurian species follow.

#### 3.1.1 Sphenodon

The heart of tuatara (*Sphenodon punctatus*) had a triangular shape with two atria with interatrial septum, and one undivided ventricle. The heart had well‐developed atrioventricular canal as well as a single atrioventricular valve, which was connected with interatrial septum. The inner heart morphology showed clearly developed trabeculae and the compact layer. MicroCT revealed well organized trabeculae, muscular ridge, and bulbuslamelle, but there was no distinct vertical septum. The heart was much more spongy than compact, but the compact layer was present and equally distributed along the ventricle. We observed also the conus arteriosus, which referred to primitive heart feature in lepidosaurs, and small muscular ridge.

#### 3.1.2 Salvator

In Argentine black and white tegu (*Salvator merianae*), the microCT revealed well‐developed three prominent ventricular septa. The main differences between varanids and tegu were found in the level of the septal prominence, e.g. in size of the muscular ridge—smaller than in varanids, and in the thickness of the compact layer. Compact layer was relatively thin in tegu similarly to *Pogona* in comparison to the varanids (with exception of *Varanus acanthurus*). The compact layer had even distribution along the ventricle. The vertical septum prominence ratio was similar to *V. griseus* and *Lanthanotus*.

#### 3.1.3 Pogona

Central bearded dragon (*Pogona vitticeps*) had a very broad heart, with well‐developed trabeculae resembling ventricle compartmentalization. The ventricle was spongious and the thin compact layer was distributed evenly along its perimeter. The bulbuslamelle and muscular ridge were present. *Pogona* was close to *Sphenodon* and *Shinisaurus* when comparing the thickness of the compact layer and the ventricular septa prominence.

#### 3.1.4 Shinisaurus

The heart of Chinese crocodile lizard (*Shinisaurus crocodilurus*) resembled *Sphenodon* heart in terms of the shape and trabeculation, but it did not have the prominent conus arteriosus. The compact layer was distributed evenly along the ventricle. Although ventricular septa were observed, their prominence was very low in comparison to other anguimorphs and it was similar to *Pogona* and *Sphenodon*.

#### 3.1.5 Heloderma

In Gila monster (*Heloderma suspectum*), the heart was broad with distinct compact layer as well as three developed ventricular septa. The compact layer was not distributed evenly and the thickness was greater on the left side. Moreover, compact layer thickness ratio was closer to varanids and *Lanthanotus*. Prominence of the vertical septum ratio showed closeness to *Sphenodon* and *Salvator*.

#### 3.1.6 Lanthanotus

In Borneo earless monitor (*Lanthanotus borneensis*) were observed some of the characteristics as in varanids: good ventricular compartmentalization and well‐developed compact layer. The compact layer was evenly distributed along the ventricle similarly to tegu but not like in varanids, where the compact layer was thicker in the left part of the ventricle. The prominence of the vertical septum ratio showed clearly that *Lanthanotus* heart had a good septation of the ventricle.

#### 3.1.7 Varanus sp.

In Argus monitor (*Varanus panoptes horni*), the heart weight was 15 g, width 4 cm, and height 4.5 cm of female specimen, total weight 2.5 kg. The hearts had a well‐developed ventricular compartmentalization by three septa. Towards the apex, the muscular ridge and the bulbuslamelle were merged and formed a single septum that separated the cavum pulmonale from the left side of the ventricle. The compact layer showed difference in distribution between the right and left ventricular part in favor of the left part of the ventricle. However, the microCT of the heart morphological characteristics revealed differences among the chosen monitor lizards, more specifically in the distribution level of the compact layer and the vertical septum prominence. The most prominent vertical septum ratio was observed in Desert monitor (*Varanus griseus*) as well as the largest difference between the left and right part of the ventricle among all species examined, not only in the varanids.

## 4 DISCUSSION

The heart collection, as it was presented in this study, contains rare specimens, such as *Sphenodon* and *Lanthanotus*. The main limitation lied in availability of only one heart sample for each specimen (two hearts in case of *Lanthanotus*). Therefore, the measurements are proportional and no statistical evaluation could be performed. Presumably different levels of the ventricle contraction must be taken into consideration as well. The heart contraction goes together with the state of end‐systole (hearts were shrunk to minimal volume) and it is generally connected to cardiac shunts occurrence in reptiles (Burggren, 1987; Burggren et al., 2020; Hicks et al., 1996; Hicks & Wang, 1996). The cardiac shunts are very effective in reduction of the aerobic metabolism and therefore help in increasing or decreasing the metabolic demands under different conditions (Burggren et al., 2014; Wang et al., 1997) such as anoxia in turtles during wintering or diving (Hicks & Farrell, 2000), exigent exercise such as diving in crocodylians (Axelsson et al., 1996), or in lizards during dealing with hypoxia by lowering body temperature (Hicks & Wood, 1985). However, the role of cardiac shunts in varanids with double‐pump system throughout systole (Burggren & Johansen, 1982) is not yet fully understood (Heisler et al., 1983).

Our observations of the *Sphenodon* heart correlate with the findings known from the literature (Greil, 1903; Meinertz, 1966; O'Donoghue, 1921; Simons, 1965)—presence of muscular ridge, bulbuslamelle, trabeculae, and cava. We point to the fact that the compact layer is also present. In summary, tuatara's heart resembled primitive state by presence of substantial conus arteriosus (Jensen et al., 2014; Simons, 1965). In *Sphenodon*, we observed thicker spongious layer organized to trabeculae as in other ectothermic vertebrates, such as *Xenopus* (Sedmera et al., 2003). Different ventricular septation levels are derived from such primitive state in lepidosaurian heart morphology. *Pogona* resembled primitive state closely to *Sphenodon* and *Shinisaurus* despite all that bearded dragon, tuatara, and Chinese crocodile lizard fill a different phylogenetic position and ecological niche. However, all these three species are mostly sit‐and‐wait predators (Cree, 2014; Köhler et al., 2003; Ziegler et al., 2008), which is reflected in similar primitive heart morphology in terms of compact layer thickness: very thin; ventricular septation: poorly developed; and spongious layer: well‐developed and organized in trabeculae. On the other hand, in active foragers used in this study, genus *Varanus* and *Salvator* (Srbek‐Araujo et al., 2020; Thompson & Withers, 1997), we found the opposite situation in heart morphology. The compact layer was thick and the ventricular septation was well‐developed by three incomplete ventricular septa. Nevertheless, the difference between *Varanus* and *Salvator* lies in thickness and distribution of the compact layer and size of the muscular ridge. The thickness and distribution of compact layer varies among these two groups, especially when compared with desert and argus monitors. The varanids compact layer is thicker and distributed in favor of the left part of the ventricle, contrary to tegu, where the compact layer is thin and evenly distributed along the ventricle similarly to other typical lizards used in this study. The size of muscular ridge is as in a typical lizard as well. The ventricular septa are also less prominent, especially in size of muscular ridge in *Salvator* compared with hat in *Varanus* referred to the differences in the metabolism and ecological niche. Besides these two factors, there is a significant connection between cardiovascular system and lungs morphology and development: single‐chambered in *Salvator*, and multi‐chambered in *Varanus* with double respiratory surface area in comparison to *Salvator* (Perry, 1998). The double sized respiratory surface area helps with high aerobic performance in varanids (Wood et al., 1978), whereas such performance in tegus is much lower (Toledo et al., 2008) and it is also affected with great seasonal effect (Sanders et al., 2015). Multi‐chambered lungs were also found in *Heloderma*, which is a unique feature among lepidosaurs (Perry, 1998). Heart of *Heloderma* showed features typical for varanids (thicker compact layer distribution in favor of the left part of the ventricle) but also features for a typical lizard (low prominence of the vertical septum). However, the variations in heart structure and physiology do not have to be necessarily connected to lepidosaurian lung morphology. This state can be demonstrated in sister taxa Pythonidae and Boidae (Noonan & Chippindale, 2006; Reynolds et al., 2014), where the lungs morphology is similar (Brongersma, 1951; Perry, 1998) but there are differences in the heart morphology such as ventricular septation in pythons but not in boas (Jensen et al., 2014), and also in blood pressure: mammalian‐like pressure in pythons in contrast to boas (Wang et al., 2001; Zaar et al., 2007). Vascularized compact layer plays a key role too. The ventricular compact layer helps in better blood ejection to the body and it is present in all amniotic vertebrates (Bettex et al., 2014) as well as in some fish (Farrell et al., 2012; Simões et al., 2002), and in some amphibians (Jewhurst & McLaughlin, 2015) such as in Greater Siren (*Siren lacertina*; Putnam, 1977).

### 4.1 Functional heart morphology and blood streams separation

As it was mentioned, all lepidosaurs have trabeculae, compact layer, and three functional incomplete septa (vertical septum, bulbuslamelle, and muscular ridge). These structures help in separation of oxygenated and deoxygenated blood in the ventricle (Jensen, Nielsen, et al., 2010; Millard & Johansen, 1974; Starck & Wyneken, 2022; Webb et al., 1971). In case of lepidosaurs, the nocturnal animals with low metabolism such as *Sphenodon* (Thompson & Daugherty, 1998), *Heloderma* (Beck & Lowe, 1994) or *Lanthanotus* (Pianka & King, 2004) use the trabeculae and also the septa (*Heloderma, Lanthanotus*) for better blood separation typically for ectotherms with low metabolism (Johansen & Hanson, 1968; Olejnickova et al., 2021; Sedmera et al., 2003). Similarly, diurnal sedentary (sit‐and‐wait) animals, *Shinisaurus* (Ziegler et al., 2008) and *Pogona* (Köhler et al., 2003) also use both—trabeculae and ventricular septa, which are, however, not so prominent as in varanids. On the other hand, athletic diurnal animals, varanids (Clemente et al., 2009; Thompson & Withers, 1997) or *Salvator* (Ferreguetti et al., 2018; Toledo et al., 2008), use mainly the septal ventricular structures for blood separation (Johansen & Burggren, 1984; White, 1968). These structures compartmentalize the lepidosaurian ventricle into three cava (cavum venosum, pulmonale, and arteriosum) which also could help in the blood streams separation (Jensen et al., 2014; White, 1968). A difference between varanids and *Salvator* was found in compact layer distribution and thickness, which was more massive in monitor lizards, connected also with their mammalian‐like high blood pressure, approx. 110/80 mmHg (mean arterial pressure 12 kPa), (Burggren & Johansen, 1982; Heisler et al., 1983) as a key adaptation in monitor lizards (Hanemaaijer et al., 2019; Thompson & Withers, 1997). Such blood pressure was not observed in Teiidae (Skovgaard et al., 2005). Blood pressure in tegu is approx. 50/37 mmHg (mean arterial pressure 5.66 kPa; Filogonio et al., 2020). The blood pressure is going hand in hand with high seasonal variability in metabolism as well as with ontogenetic shift and life‐histories not only in large tegu species (Piercy et al., 2015; Toledo et al., 2008) but also in the whole Teiidae family as it was observed in genus *Ameiva* (Morgan, 1988). Temperature is another key player, which influences the cardiovascular system in squamates, especially in connection with particular ecological niche.

### 4.2 Impact of the temperature on cardiovascular system as an example of significant abiotic factors

Impacts of abiotic factors to individuals or species are commonly observed across the animal kingdom. Ecological niche is reflected in animal physiology, the change is also reflected on anatomy and morphology, and cardiovascular system is not an exception. An ectothermic animal has to deal with changeable thermal conditions, which are significant for ectotherms. Large and active foragers such as tuna fish (*Thunnus orientalis*) have thermal adaptation, which allows them to expand into the new colder ecological niche and so it affects the cardiovascular system (Blank et al., 2004). Such impact includes not only the heart but also the surface of the gills and the blood circulation in the whole body (Bushnell & Brill, 1992; Bushnell & Jones, 1994). Therefore, the temperature is the key factor for the ectotherms (Huey & Stevenson, 1979) and it is also true for lepidosaurs. Hence, it is no surprising that similar observations were also demonstrated across different ectothermic species, in salmonids (Klaiman et al., 2011) or in free‐ranged lizard (*Pogona barbata*; Grigg & Seebacher, 1999). Such observations showed that the heart reacts immediately to the change and heart rates are linked to the heat exchange during heating and cooling as it was described for large monitor lizard species Lace monitor (*Varanus varius*; Seebacher & Grigg, 2001). These important findings lead to the preview of ectothermy as a specific adaptation to the thermal abiotic conditions with advantages such as saving the energy or inhabiting new ecological niches (Rodda, 2020). Therefore, we propose that the particular ecological niche is one of the dynamical drivers for changes in cardiovascular system in lepidosaurs. Moreover, our results support the idea showed in Figure 1 that the cardiovascular arrangement is dependent on the ecological niche as well as on phylogenetic position of particular species, which could be shown in model group of squamates—Anguimorpha.

### 4.3 Evolutionary view on heart morphology—Anguimorpha as a model group for studying the ventricular septation

Clade Anguimorpha shares, apart from the unified body plan, also the integrity in almost exclusively carnivorous dietary system (Pough, 1973), which goes hand in hand with evolution of the venom system (Fry et al., 2010; Koludarov et al., 2017), and with evolution of hunting, especially in varanid species (Losos & Greene, 1988). Therefore, cardiovascular system in anguimorphs covers all heart septation levels, which are encountered in connection not only with ecological niche but also to phylogenetic position. This is particularly true for the varanids. According to our results the higher phylogenetic position, the higher the loss of primitive features (presence of the conus arteriosus, small muscular ridge, well developed spongious layer to the detriment of the compact layer, and the level of the septation) in heart morphology. The trend is mostly observed in the incremental loss of the conus arteriosus, which goes with accretion of the size of muscular ridge. Moreover, there is a change in ratio of the spongious layer in favor of the compact layer, and increasing ventricular septation level. This trend could be seen across the group Anguimorpha from more primitive species such as *Shinisaurus* with typical lizard pattern, through *Heloderma* and *Lanthanotus* with advancing changes in heart morphology to crown species of genus *Varanus* with the most efficient heart among lepidosaurs. From phylogenetic point of view, varanids represent crown group among squamates (Ast, 2001; Pyron et al., 2013). Furthermore, monitor lizards are good models for studying ecophysiology, because they serve as an ecomorphological evolutional unit, which means they are unified not only in body plan (Brennan et al., 2021; Pianka, 1995; Pianka & King, 2004) but also in sharing effective metabolism (Bartholomew & Tucker, 1964). Such metabolism is similar to mammals (Hopson, 2012), especially in measuring the blood pressure (Burggren & Johansen, 1982; Johansen & Burggren, 1984; Seymour et al., 2012). In terms of the ventricular septation and mammalian‐like blood pressure, it seems that monitor lizards gain cardiovascular apomorphy, which is shared in all varanids (Hanemaaijer et al., 2019). Such apomorphy enables varanids to fill the top predator niche (Cross et al., 2020; Pianka, 1994). However, even among varanids there are differences in inhabiting particular ecological niche as could be demonstrated between *Varanus acanthurus* and *V. panoptes horni*. *V. acanthurus* settles a sedentary life‐history (Clemente et al., 2009) in contrast to *V. panoptes horni*, which is a very agile large foraging predator (Thompson & Withers, 1997). Such fine changes in niche, e.g. settled life‐history from original active forage mode (Clemente et al., 2009) are reflected in the heart morphology and elucidate the differences among varanids heart structures. Therefore, further analysis of the varanid heart morphology is needed.

### 4.4 Conclusion

In summary, heart morphology varies among the reptilian clades and ecological niches of the particular species. Settled or nocturnal animals do not need good ventricular septation. On the other hand, active foragers need to be prepared for hunting and/or changing locomotory types, e.g. from walking to running. Therefore, ventricular septa reflect well the ecological niche in better blood streams separation resulting in cardiac shunts in agile varanids with active foraging mode. The level of such ventricular septation is reflected in the phylogenetic position in clade Anguimorpha as well. Moreover, monitors, with mammalian‐like blood pressure and almost full functionally septated ventricle, need also a good blood supplement for the working myocardium. Such blood supplement is provided by thick and well vascularized compact layer, which evolved in connection with metabolic as well as ecological state. However, there is a high variety among lepidosaurs in terms of metabolism, body plans, and ecological strategies. Therefore, it is challenging to adapt the results as rule for all lepidosaurs, especially for squamates. Unique uniformity of Anguimorpha clade reveals how the transition could be changed from primitive state (*Shinisaurus*) to such derived state (*Varanus*). In the absence of fossil evidence, this is the closest approach to understanding the evolution of the heart and its septation in squamate reptiles.

## AUTHOR CONTRIBUTIONS

**Martina Gregorovičová:** Conceptualization (lead); data curation (lead); formal analysis (lead); investigation (lead); methodology (lead); project administration (lead); resources (equal); supervision (lead); validation (lead); visualization (lead); writing – original draft (lead). **Martin Bartos:** Formal analysis (equal); methodology (equal); software (lead); visualization (equal). **Bjarke Jensen:** Formal analysis (equal); investigation (equal); validation (equal); writing – original draft (supporting). **Jiri Janacek:** Formal analysis (equal); software (equal); validation (equal). **Bryan Minne:** Data curation (equal); resources (equal). **Jiri Moravec:** Data curation (equal); resources (equal); writing – original draft (supporting). **David Sedmera:** Formal analysis (equal); funding acquisition (lead); validation (equal); writing – original draft (equal).

## FUNDING INFORMATION

Progres Q38/1LF. Progres Q29/1LF. RVO: 67985823. MEYS CR: LM2018129. MEYS CR: Cooperatio 207029 Cardiovascular Sciences.

## CONFLICT OF INTEREST

Authors declare no conflict of the interest.

## Supporting information


Appendix S1

## Data Availability

Data available on request from the authors. The data that support the findings of this study are available from the corresponding author upon reasonable request. The supplementary data are available on Dryad dataset: https://doi.org/10.5061/dryad.crjdfn37k.
