# Supplementary material for: Anguimorpha as a model group for studying the comparative heart morphology among Lepidosauria: Evolutionary window on the ventricular septation
Source: Ecol Evol. 2022 Nov 8;12(11):e9476. doi: 10.1002/ece3.9476 (PMC9643144; doi:10.1002/ece3.9476)
Supplement: Supplementary file 1 — Appendix S1 [file ECE3-12-e9476-s001.docx]

Supplementary material: whole 3D models of selected lepidosaurian species.
